# A heterozygous mutation in the *CCDC88C* gene likely causes early-onset pure hereditary spastic paraplegia: a case report

**DOI:** 10.1186/s12883-021-02113-y

**Published:** 2021-02-18

**Authors:** Ashraf Yahia, Zhefan Stephen Chen, Ammar E. Ahmed, Sara Emad, Rawaa Adil, Rayan Abubaker, Shaimaa Omer M. A. Taha, Mustafa A. Salih, Liena Elsayed, Ho Yin Edwin Chan, Giovanni Stevanin

**Affiliations:** 1 Department of Biochemistry, Faculty of Medicine, University of Khartoum, Alqsr Street, Khartoum, Sudan; 2 Department of Biochemistry, Faculty of Medicine, National University, Khartoum, Sudan; 3 Institut du Cerveau, INSERM U1127, CNRS UMR7225, Sorbonne Université, Paris, France; 4 School of Life Sciences, Faculty of Science, The Chinese University of Hong Kong, Shatin, N.T., Hong Kong, SAR China; 5 Department of Physiology, Faculty of Medicine, University of Khartoum, Khartoum, Sudan; 6 Faculty of Medicine, University of Khartoum, Khartoum, Sudan; 7 Institute of Endemic Diseases, University of Khartoum, Khartoum, Sudan; 8 Department of Radiology, Dar Al Elaj Specialized Hospital, Khartoum, Sudan; 9 Division of Pediatric Neurology, Department of Pediatrics, College of Medicine, King Saud University, Riyadh, Saudi Arabia; 10 Gerald Choa Neuroscience Centre, The Chinese University of Hong Kong, Shatin, N.T., Hong Kong, SAR China; 11 Ecole Pratique des Hautes Etudes, EPHE, PSL Research University, Paris, France

**Keywords:** Spinocerebellar ataxia type 40, Hereditary spastic paraplegia, *CCDC88C*, Sudan

## Abstract

**Background:**

CCDC88C is a ubiquitously expressed protein with multiple functions, including roles in cell polarity and the development of dendrites in the nervous system. Bi-allelic mutations in the *CCDC88C* gene cause autosomal recessive congenital hydrocephalus (OMIM #236600). Studies recently linked heterozygous mutations in *CCDC88C* to the development of the late-onset spinocerebellar ataxia type 40 (OMIM #616053).

**Case presentation:**

A 48-year-old Sudanese female presented with pure early onset hereditary spastic paraplegia. Exome sequencing, in-silico analysis, and Sanger sequencing identified the heterozygous NM_001080414.4:c.1993G > A (p.E665K) variant in *CCDC88C* as a potential cause of her illness. To explore the pathogenicity of the NM_001080414.4:c.1993G > A (p.E665K) variant, we expressed it in human embryonic kidney 293 cells and assessed its effects on apoptosis. In our experiment, NM_001080414.4:c.1993G > A (p.E665K) induced JNK hyper-phosphorylation and enhanced apoptosis. In contrast to previous reports, our patient developed neurological symptoms in early childhood and showed neither features of cerebellar ataxia, extrapyramidal signs, nor evidence of intellectual involvement.

**Conclusion:**

We, herein, heighlighted the possibility of extending the phenotype associated with variants in *CCDC88C* to include early-onset pure hereditary spastic paraplegia.

**Supplementary Information:**

The online version contains supplementary material available at 10.1186/s12883-021-02113-y.

## Background

Hereditary spastic paraplegia and hereditary ataxia are distinct entities that share common clinical features and pathological mechanisms [1, 2]. Bi-allelic mutations in the *CCDC88C* gene are known to cause autosomal recessive non-syndromic congenital hydrocephalus (OMIM # 236600) [3]. Furthermore, two reports recently linked heterozygous mutations in *CCDC88C* to the development of the autosomal dominant spinocerebellar ataxia type 40 (SCA40; OMIM # 616053) [4, 5]. In the first report, the variant NM_001080414.4:c.1391G > A (p.R464H) caused a phenotype of ataxia, dysarthria, and pyramidal features, brisk reflexes in one patient and spastic lower limbs in a second patient, in a family from Hong Kong [4]⁠. In the second report, the variant NM_001080414.4:c.127G > A (p.D43N) caused a phenotype of ataxia with parkinsonian features and dementia in a Polish family [5]. The age at onset in the patients from both families, when provided, was above 30 years [4, 5]. The severity of the ataxia ranged from severe in the Chinese family to relatively mild in the Polish family. However, unlike in the Chinese patients, the phenotype in the Polish family involved extrapyramidal features [4, 5].

We, herein, report for the first time a case of pure childhood-onset hereditary spastic paraplegia caused by the heterozygous variant NM_001080414:c.1993G > A (p.E665K) in the *CCDC88C* gene.

## Case presentation

**Fig. 1:**
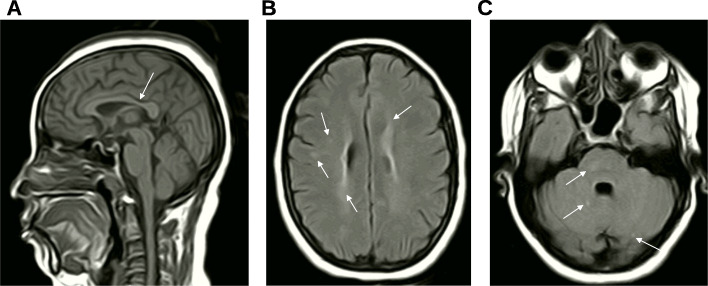
Brain MRI of the patient F83–581. **a** Sagittal T1 section showing thinning in the isthmus of the corpus callosum (arrow). **b** and **c** Axial T1 section showing periventricular white matter changes and scattered hyperintensities in the white matter, cerebellum, and right side of the pons (arrows). The cerebrum, brain stem, and cerebellum were not atrophied

A 48-year-old Sudanese lady, coded as F83–581, presented with an abnormal gait as a manifestation of pure hereditary spastic paraplegia. Her condition started in early childhood with tip-toeing that progressed gradually in severity. At the age of 30 years, she could walk only using two sticks. She did not complain of any additional symptoms apart from occasional muscle cramps. Her parents were distantly related and had no family history of similar conditions. She was not on treatment. On examination, her lower limbs were spastic with severe weakness (power grade 3). There were bilateral deformities in the feet (pes equinovarus on the right and hammertoe on the left) and up-going plantar responses. Her upper limbs were normal except for mild spasticity and hyperreflexia on the right side. The patient (F83–581) had neither signs of cerebellar involvement nor evidence of sensory deficit. She was cooperative, oriented, and had no evidence of intellectual alteration. She could barely walk supported by two sticks, and her gait was spastic. Nerve conduction studies were normal. Brain magnetic resonance imaging (MRI) showed periventricular leukomalacia with scattered ischemic foci in the white matter, cerebellum, and right side of the pons. The isthmus of the corpus callosum was thin, but it could be a normal variant. We noted neither cerebral, brain stem, nor cerebellar atrophy, nor acute ischemic changes on the brain MRI (Fig. 1).

**Fig. 2:**
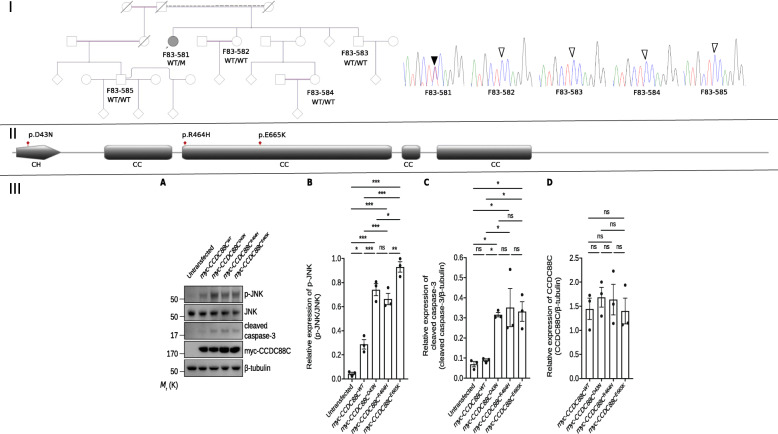
Segregation analysis, schematic representation of CCDC88C protein, and functional validation of the variant NM_001080414.4:c.1993G > A (p.E665K). I. The family pedigree and chromatogram showing the mutant variant NM_001080414.4:c.1993G > A (p.E665K) (filled arrowhead) heterozygous in the patient F83–581 (filled circle) and the wild-type variant at the same position (empty arrowheads) homozygous in the healthy controls (coded circles and square). M: mutant allele; WT: wild type allele. II. Schematic representation of CCDC88C protein based on the Uniprot entry Q9P219 showing the locations of the reported pathogenic heterozygous variants in the protein. CC: coiled-coil region; CH: calponin-homology domain. III. Expression of CCDC88C^E665K^ mutant protein activated c-Jun N-terminal kinase (JNK) / caspase-3 apoptotic signaling pathway. **a** The wild-type and mutant *pcDNA3.1(+)-myc-CCDC88C* expression constructs were used to transfect Human Embryonic Kidney 293 (HEK293) cells to assess the effect of CCDC88C^E665K^ mutant protein on the activity of JNK / caspase-3 signaling pathway. Similar to the other two CCDC88C mutant proteins (CCDC88C^D43N^ and CCDC88C^R464H^) that were previously reported to activate JNK / caspase-3 signaling [4, 5], overexpression of CCDC88C^E665K^ mutant protein caused a significant increase of JNK phosphorylation and caspase-3 cleavage. *n* = 3 biological replicates on 3 independent preparation of protein samples. Only representative blots were shown. **b-d** Quantification of the relative expression of p-JNK (**b**), cleaved caspase-3 (**c**), and CCDC88C (**d**) proteins. Error bars represent S.E.M. Statistical analysis was performed using one-way ANOVA followed by post hoc Tukey’s test. ns denotes no significant difference, * denotes *p* < 0.05, ** denotes *p* < 0.01 and *** denotes *p* < 0.001

We extracted DNA from the patient and four of her family members and investigated the patient and one of her healthy siblings, coded F83–582, using whole-exome sequencing [6]. Whole-exome sequencing of the patient revealed a heterozygous variant, NM_001080414.4:c.1993G > A (p.E665K) (rs956104232), in the *CCDC88C* gene that results in substituting Glutamate at position 665 of the protein for Lysine. Sift [7], Polyphen2 HDIV [8], Mutation Taster [9], Provean [10] and M-cap [11] embedded in VarAFT software [12] predicted this substitution as pathogenic with prediction scores of 0.002, 0.982, 1, − 3.21 and 0.069, respectively. Glutamate at position 665 of CCDC88C is highly conserved during evolution. The CADD score of 25 was also in favor of a pathogenic role of this change. We did not detect other convincing variants that could explain the phenotype in our patient. The variant NM_001080414.4:c.1993G > A (p.E665K) was reported once in the gnomAD v2.1.1 database in an individual of African ancestry and had a global allele frequency of 0.0000032 [13]. Using Sanger sequencing, we validated that the variant NM_001080414.4:c.1993G > A (p.E665K) was heterozygous in the patient and absent in her healthy family members (Fig. 2-I).

To validate the pathogenicity of the NM_001080414.4:c.1993G > A (p.E665K) variant, we expressed the CCDC88C cDNA in human embryonic kidney (HEK) 293 cells and assessed its effect on c-Jun N-terminal kinase (JNK) / caspase-3 signaling pathway according to the presence or absence of the variant. Overexpressing CCDC88C^E665K^ mutant protein caused a significant increase of JNK hyperphosphorylation and caspase-3 cleavage compared to the wild type protein, a pattern also seen when overexpressing the known SCA40 pathogenic proteins CCDC88C^D43N^ and CCDC88C^R464H^ (Fig. 2-III and supplementary data [6]). NM_001080414.4:c.1993G > A (p.E665K) was likely a de novo variant, though we did not have DNA samples from the parents. It had a low frequency in gnomAD database, predicted as pathogenic by multiple computational tools, and its pathogenicity was corroborated by functional studies, thus, fulfilling the criteria of likely pathogenic variants according to the American college of medical genetics and genomics guidelines for interpreting sequence variants published in 2015 [14]. We have submitted the variant to the Clinvar database (accession VCV000978819.2).

## Discussion and conclusion

CCDC88C is a cytoplasmic protein ubiquitously expressed in mammals and has a variety of functions [15]. Up-to-date, mutations in CCDC88C are linked to monogenic neurological phenotypes by one of two mechanisms depending on the mutant allele, activation of hetero-trimeric G-proteins [16] and activation of JNK cascade [4].

Bi-allelic loss of function mutations in *CCDC88C* cause autosomal recessive congenital hydrocephalus (OMIM #236600) by disrupting the morphogenesis of the nervous system [3, 16]. On the other hand, gain-of-function mutations in *CCDC88C* cause SCA40 through mechanisms that induce JNK hyper-phosphorylation and prompt apoptosis [4].

In this report, we showed that a mutation in the *CCDC88C* gene induces JNK hyper-phosphorylation and the resulting activation of apoptosis in early-onset pure hereditary spastic paraplegia. Recently the borders between hereditary ataxia and hereditary spastic paraplegia have been questioned [2]. SCA40 is an emerging clinical entity identified up-to-date in only two families worldwide (Table 1), the first family was from China and the second family was from Poland [4, 5]. Cerebellar features were present in both families, though with different degrees of severity. Extrapyramidal features and dementia dominated the phenotype in the Polish family. Pyramidal involvement, hyperreflexia in one patient and spasticity in a second patient, was only evident in examining the Chinese family’s patients. The pathogenic mutation in the Polish family, NM_001080414.4:c.127G > A (p.D43N), is located in the calponin-homology domain of the CCDC88C protein. The mutations reported in our patient and in the Chinese patients, NM_001080414.4:c.1993G > A (p.E665K) and NM_001080414.4:c.1391G > A (p.R464H), respectively, are located in a coiled-coil domain in the protein (Fig. 2-II).

**Table 1:** Summary of the reported clinical phenotypes of the patients with pathogenic mutations in the *CCDC88C* gene

Patient	II:4	II:5	IV-2	IV-1	IV-3	III-3	F83–581
Reference	(Tsoi et al., 2014) [4]		(Leńska-Mieciek et al., 2019) [5]				This report
Origin	China		Poland				Sudan
Gender	Female	Male	Male	Female	Male	Female	Female
Age	65 years	62 years	59 years	63 years	53 years	84 years	48 years
Age at onset	43 years	42 years	49 years	45 years	33 years	–	Early childhood
Presentation	Ataxic gait and dysarthria	Ataxic gait and dysarthria	Rest and action upper limbs tremors	Rest and action right upper limb tremors	Rest and action upper limbs tremors	Rest and action upper limbs and head tremors	Abnormal gait (spastic)
Signs on examination							
Cerebellar ataxia	Yes	Yes	Yes	Yes	Yes	–	No
Hyper-reflexia	Yes	Yes	Yes	–	–	–	Yes
Spasticity	–	Yes	–	–	–	–	Yes
Extra-pyramidal features	No	No	Rigidity and bradykinesia	Bradykinesia	Rigidity and bradykinesia	–	No
Others	–	Opthalmo-plegia	Cognitive impairment	Cognitive impairment	Cognitive impairment	Cognitive impairment	Lower limbs weakness
SARA score	24/40	22/40	10/40	5/40	3/40	–	–
Brain MRI	Ponto-cerebellar atrophy, subcortical T2 hyper-intensities	Moderate ponto-cerebellar atrophy, subcortical T2 hyper-intensities	Normal	Normal	Normal	–	Periventricular leukomalacia with scattered ischemic foci
Nerve conduction studies	–	–	–	–	–	–	Normal

Although we did not have DNA samples from the patient’s parents, we assumed NM_001080414.4:c.1993G > A (p.E665K) was a de novo variant because the parents neither manifested the disease throughout their lives nor had a positive family history. However, we could not completely rebut the possibility of incomplete penetrance in the parents. The early age at onset in our patient compared to the two previously reported SCA40 families, adds another layer of complexity and suggests the presence of unknown modifiers. More studies are needed to delineate the phenotypes’ spectrum associated with *CCDC88C* mutations and investigate genotype-phenotype correlations.

In conclusion, this report suggests the extension of *CCDC88C*-associated phenotype to include early-onset pure hereditary spastic paraplegia.

## Supplementary Information

**Additional file 1.**

## Data Availability

Supplementary data for this study are openly available in Figshare repository at 10.6084/m9.figshare.13020581.v1 [6].

## Abbreviations

SCA40: Spinocerebellar ataxia type 40
OMIM: Online inheritance in man
MRI: Magnetic resonance imaging
JNK: c-Jun N-terminal kinase
HEK: Human Embryonic Kidney
