# Supplementary material for: A heterozygous mutation in the CCDC88C gene likely causes early-onset pure hereditary spastic paraplegia: a case report
Source: BMC Neurol. 2021 Feb 18;21:78. doi: 10.1186/s12883-021-02113-y (PMC7890981; doi:10.1186/s12883-021-02113-y)
Supplement: Supplementary file 1 — Additional file 1. [file 12883_2021_2113_MOESM1_ESM.pdf]

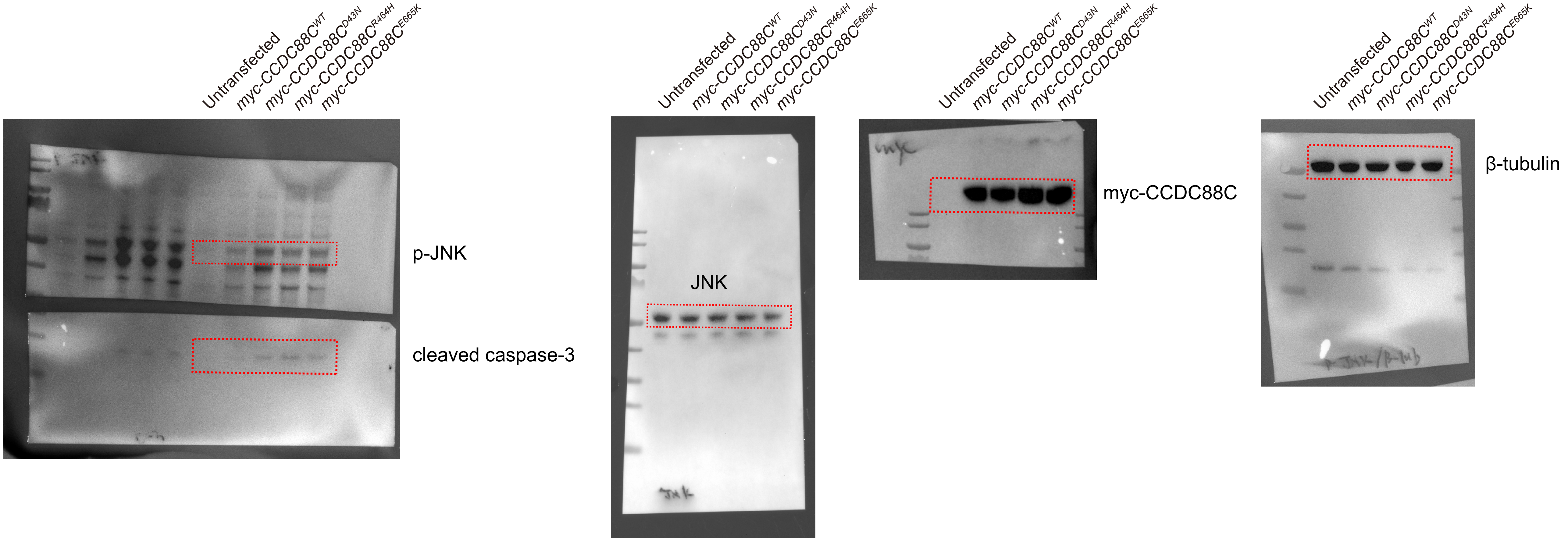
Untransfected
myc-CCDC88C^{WT}
myc-CCDC88C^{D43N}
myc-CCDC88C^{R464H}
myc-CCDC88C^{E665K}
p-JNK
cleaved caspase-3
JNK
myc-CCDC88C
β-tubulin
